# Simultaneous Quantification of Antioxidants Paraxanthine and Caffeine in Human Saliva by Electrochemical Sensing for CYP1A2 Phenotyping

**DOI:** 10.3390/antiox10010010

**Published:** 2020-12-24

**Authors:** Rozalia-Maria Anastasiadi, Federico Berti, Silvia Colomban, Claudio Tavagnacco, Luciano Navarini, Marina Resmini

**Affiliations:** 1 Department of Chemistry, Queen Mary University of London, Mile End Road, London E1 4NS, UK; 2 Department of Chemical and Pharmaceutical Sciences, University of Trieste, via L. Giorgieri 1, 34127 Trieste, Italy; fberti@units.it (F.B.); c.tavagnacco@units.it (C.T.); 3 Aromalab, illycaffè S.p.A., Area Science Park, Localita’ Padriciano 99, 34149 Trieste, Italy; silvia.colomban@illy.com (S.C.); luciano.navarini@illy.com (L.N.)

**Keywords:** paraxanthine, caffeine, CYP1A2 phenotyping, antioxidants, human saliva, differential pulse voltammetry

## Abstract

The enzyme CYP1A2 is responsible for the metabolism of numerous antioxidants in the body, including caffeine, which is transformed into paraxanthine, its main primary metabolite. Both molecules are known for their antioxidant and pro-oxidant characteristics, and the paraxanthine-to-caffeine molar ratio is a widely accepted metric for CYP1A2 phenotyping, to optimize dose–response effects in individual patients. We developed a simple, cheap and fast electrochemical based method for the simultaneous quantification of paraxanthine and caffeine in human saliva, by differential pulse voltammetry, using an anodically pretreated glassy carbon electrode. Cyclic voltammetry experiments revealed for the first time that the oxidation of paraxanthine is diffusion controlled with an irreversible peak at ca. +1.24 V (vs. Ag/AgCl) in a 0.1 M H_2_SO_4_ solution, and that the mechanism occurs via the transfer of two electrons and two protons. The simultaneous quantification of paraxanthine and caffeine was demonstrated in 0.1 M H_2_SO_4_ and spiked human saliva samples. In the latter case, limits of detection of 2.89 μM for paraxanthine and 5.80 μM for caffeine were obtained, respectively. The sensor is reliable, providing a relative standard deviation within 7% (*n* = 6). Potential applicability of the sensing platform was demonstrated by running a small scale trial on five healthy volunteers, with simultaneous quantification by differential pulse voltammetry (DPV) of paraxanthine and caffeine in saliva samples collected at 1, 3 and 6 h postdose administration. The results were validated by ultra-high pressure liquid chromatography and shown to have a high correlation factor (r = 0.994).

## 1. Introduction

Caffeine is probably one of the most studied methylxanthines to date and is also one of the early examples of functional food ingredients, with multiple studies reporting on the significant health benefits associated with caffeine (CAF) consumption [1]. The roles of CAF as central nervous stimulant, antioxidant [2,3], pro-oxidant [4], cognitive enhancer [5] and neuroprotectant [6,7] are all well documented. An interesting physiological effect of CAF derives by acting as an adenosine analogue, blocking the adenosine receptors [8], and resulting in increased release of hormones, with an overall neuroprotective effect [9]. This has also sparked interest towards the development of new adenosine and caffeine analogues [10]. Furthermore, the consumption of CAF has recently been shown to reduce oxidative DNA damage and prevent lipid peroxidation [11], leading to suggestions that its consumption could positively impact diseases such as type 2 diabetes, depression, and liver, Parkinson’s [12] and Alzheimer’s disease [13,14].

**Figure 1:**
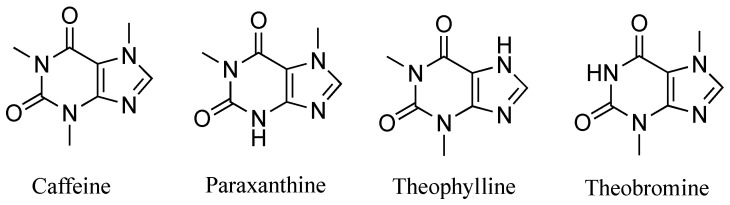
Chemical structures of caffeine and its primary metabolites.

In the human body, CAF is metabolised more than 90% by the enzyme CYP1A2 and selectively catalysed to paraxanthine (PX), its major primary metabolite (81.5%), and theobromine (TB) and theophylline (TP) are found in 10.8% and 5.4%, respectively (Figure 1) [8]. 

These metabolites share many of the physiological activities of CAF including its antioxidant and pro-oxidant properties, with PX and TP showing a higher affinity towards the adenosine receptors compared to CAF. Even though TB and TP are found in food sources, PX is present in the body only as a result of the metabolism of CAF [15].

The human CYP1A2 enzyme is also responsible for the metabolism of more than 200 clinically important drugs including antidepressants, antipsychotics, anticancer and analgesic drugs, with many of them also having antioxidant properties [16,17,18,19,20]. However, the activity of CYP1A2 shows significant interindividual variability (10- to 200-fold) that seriously influences drugs’ metabolism and efficacy [21,22,23]. This highlights the importance of being able to evaluate the metabolic activity of CYP1A2 as a useful tool to optimise the drug dose–response effect and effectively monitor drug therapy [24,25]. This of course also impacts the metabolism of caffeine and any pharmaceutically active analogues. Considerable progress has been made in predicting drug pharmacokinetics and response in individuals by CYP1A2 phenotyping, but its application in a routine clinical setting remains a challenge [26]. Currently, CAF represents the only fully validated probe for CYP1A2 phenotyping [25,27]. The quantification of the PX-to-CAF (PX/CAF) molar ratio, at a single time-point, 4 to 8 hours upon oral administration of a 100–200 mg CAF dose is an accepted standard in clinical practice [24,25,27,28], although it currently relies on the use of highly specialised equipment, such as high pressure liquid chromatography. In terms of biological matrix, blood, plasma or urine have been traditionally used for CYP1A2 phenotyping; however, the blood and plasma required specialised personnel for sample collection, while drugs’ concentrations in urine tend to have a much higher variability and are seldom considered to be reliable.

Saliva has been investigated as an alternative sampling matrix for CYP1A2 phenotyping since it reflects 65–85% of plasma kinetics and shows close correlation with the immunoreactive CYP1A2 liver intrinsic activity [24,29,30]. This makes saliva a more viable approach, being non-invasive, patient-friendly, easier to acquire and it also allows the sampling of patients outside of a clinical setting, and for this reason was selected in this work [31,32]. To date, CAF and PX have been quantified simultaneously using high-performance liquid chromatography (HPLC) coupled with an ultraviolet (UV) detector [33,34,35,36,37], or, to a minor extent, with a mass spectrometer (MS) [38,39]. Although these techniques provide indubitable selectivity and sensitivity, they require costly and heavy equipment, and trained personnel, in addition to being non-environmentally friendly given the large amounts of solvents used. There is an urgency for alternative robust, cheaper, portable and easy-to-operate techniques with high throughput capabilities.

Electrochemical sensors are a valid alternative that allow point-of-care testing and nowadays are attracting increased interest for routine drug analysis of biomarkers [40]. Electrochemical techniques can be used to study the redox behaviour and therefore the antioxidant capacity, as well as quantification [41,42]. Carbon-based electrode materials are ubiquitous in electroanalytical laboratories due to their broad applicability for numerous molecules, wide potential window, low cost, low background current, chemical inertness and feasibility for modification, with glassy carbon (GC) being one of the most commonly used electrodes for analytical purposes, with a good chemical stability and satisfactory potential window [43,44]. Recent efforts on further improving their analytical performance have been reported using a number of modifying materials (e.g., carbon nanomaterials [45], imprinted polymers [46] and enzymes [47]) and their combinations. Despite the improvements on the sensitivity as a result of modifications, their fabrication requires additional steps and higher costs, the former potentially leading to lower reproducibility, short-term stability and challenges when used with biofluids. Despite the significant amount of literature data on modified electrodes [48], the use of bare electrodes still represents the simplest possible approach for electrochemical sensing given the commercial availability and its long-term stability.

Nowadays, electrochemical sensing using human saliva as matrix is attracting a lot of interest [49,50]. A number of studies have been reported on the detection in human saliva of important biomarkers such as uric acid [51,52], cortisol [53] and selegiline [54] using a variety of solid and screen printed carbon electrodes. Although some work has been published on the detection of CAF and other dimethylxanthines (PX, TP and TB) in biofluids such as urine and blood [55,56], beverages [57], food [58] and pharmaceutical formulations [59], the use of saliva as matrix has yet to be evaluated. Using human saliva as matrix brings a major challenge associated with the complexity of its composition and the combination of large and small molecules present; this is further complicated by the interpersonal variability observed among individuals, due to genetics, nutrition and habits [32,60]. 

Here, we report, for the first time, the simultaneous quantification of antioxidants PX and CAF in human saliva by differential pulse voltammetry (DPV) in aqueous media, employing an anodically pretreated GC electrode. The oxidation behaviour of PX was also investigated by cyclic voltammetry (CV) on the GC electrode. The experimental parameters of the DPV method were optimised using equimolar mixtures of PX and CAF, their analytical performance was evaluated and the assay was validated using artificially spiked human saliva. Qualitative assessment using a boron-doped diamond (BDD) and a screen-printed carbon paste (SP-CP) electrode was also performed for comparison purposes. Preliminary pharmacokinetic studies of CAF were carried out on five healthy human volunteers in order to provide initial evidence of the suitability of the sensing platform. The PX/CAF metabolic ratio was determined at various time intervals by DPV, validating the data with ultra-high pressure liquid chromatography coupled with ultraviolet detector (UHPLC-UV). The developed electrochemical system provides a new insight on designing efficient and robust platforms for enzyme metabolic phenotyping as personalised medicine tools. 

## 2. Materials and Methods 

### 2.1. Chemicals

Caffeine (CAF, ≥99%), paraxanthine (PX, ≥98%), theophylline (TP, ≥99%), theobromine (TB, ≥98%) and boric acid (≥95%), purchased from Sigma-Aldrich Chemie GmbH (Steinheim, Germany), were used as received. Sulphuric acid (H_2_SO_4_, 95–98%), phosphoric acid (H_3_PO_4_, ≥85%), nitric acid (HNO_3_, ≥65%), acetic acid (≥99%), formic acid (≥95%), sodium hydroxide (NaOH, ≥99%), ethanol, acetonitrile and ethyl acetate were obtained from Sigma-Aldrich with analytical grade purity. Potassium chloride (KCl, ≥3 M) solution for Ag/AgCl reference electrodes was purchased from Sigma-Aldrich. PROPLUS tablets, with a declared amount of 50 mg CAF per tablet, were obtained from a local pharmacy in London, UK. Ultra-pure deionised water (resistivity not lower than 18 MΩ cm at 25 °C) obtained from a Milli-Q unit (Millipore) was used to prepare all the solutions. 

### 2.2. Apparatus

Voltammetric measurements were conducted in a conventional glass cell using an μSTAT 400 potentiostat controlled by DropView 8400 software (DropSens, Oviedo, Spain). A three-electrode setup was used, consisting of a working glassy carbon (GC) electrode with an active surface of 5 mm diameter, a platinum counter electrode and a Ag/AgCl (3 M KCl) reference electrode, chemically isolated from the test solution with a porous Vycor frit (Metrohm Autolab B.V., Utrecht, The Netherlands). A boron-doped diamond (3 mm diameter, BDD, Windsor Scientific Ltd., Slough, UK) and disposable screen-printed carbon paste (SP-CP, model DRP-110, 4 mm diameter, DropSens, Oviedo, Spain) were also used as working electrodes.

The UHPLC system consisted of a 1290 Infinity LC system equipped with an auto-sampler, a degasser, a quaternary pump, a column thermostat and a diode array detector (DAD) operating at 273 nm. A Kinetex XB-C18 2.1 mm × 100 mm, 2.6 μm column (Phenomenex Torrance, CA, USA) was used at room temperature. For the analysis of the chromatograms, LC OpenLab was used. All equipment was purchased from Agilent Technologies (Waldbronn, Germany).

### 2.3. Preparation of Working Standards

Stock solutions of CAF (5 mM), PX (5 mM), TP (5 mM) and TB (2.5 mM) were prepared by dissolving the appropriate amount in Milli-Q water or 0.1 M H_2_SO_4_ for construction of the calibration curves. All stock solutions were prepared and stored in volumetric flasks. Supporting electrolyte solutions (H_2_SO_4_, H_3_PO_4_ and HNO_3_) were prepared at concentrations in the range 0.01–0.8 M. Britton–Robinson buffer (BR) solutions were prepared at a 0.1 M concentration, in different pHs (2–10), by mixing acetic acid (40 mM), boric acid (40 mM) and H_3_PO_4_ (40 mM) in Milli-Q water and the pH was adjusted using a 0.2 M NaOH solution. All stock solutions were stored at 4 °C for a maximum of 4 weeks. 

### 2.4. Electrochemical Measurements

The GC and BDD electrodes were mechanically cleaned using an aqueous slurry of 0.05 μm alumina oxide powder on a polishing cloth pad to a mirror-like finish and rinsed thoroughly with Milli-Q water. Before use, all electrodes were electrochemically conditioned, in a 0.1 M H_2_SO_4_ solution, using cyclic voltammetry (CV) between −1.0 V and +1.8 V at a scan rate of 100 mV s^−1^ (5 cycles).

CV was used for evaluation of the electrochemical behavior of PX and CAF as an equimolar mixture in different supporting electrolytes and for assessment of the oxidation behavior (pH and scan rate studies) of PX, in a 0.1 M H_2_SO_4_ solution, on the GC electrode. Differential pulse voltammetry (DPV) was employed for assessing the analytical performance of PX and CAF and for their quantitative assessment in human saliva upon optimisation of the DPV operating parameters such as pretreatment potential and time, scan rate and step potential. Standard calibration curves were constructed by successive addition of PX and CAF solutions, individually or simultaneously, into the electrochemical cell containing 4 mL of 0.1 M H_2_SO_4_. The calibration curve of the individual analytes was obtained by increasing the concentration of either PX or CAF from 1 μM to 300 μM while the other analyte remained fixed at 10 μM (Figures S1 and S2). A combined calibration curve was obtained by simultaneously increasing both analytes from 1 μM to 200 μM (1, 2, 5, 8, 20, 40, 60, 80, 120, 150, 180 and 200 μM), using equimolar mixtures. The peak area (charge, *Q*) of the analytes was estimated by straight lines connecting the minima and maxima before and after the peak, respectively, without applying any background correction. All measurements were performed in triplicates and the average charge values were plotted against the concentration. 

### 2.5. Assay Validation for Real Application

Matrix-matched calibration curves were constructed using saliva samples obtained from a healthy volunteer who abstained from methylxanthine-containing products for 60 h. Nine saliva samples (10 mL) were collected, one was used as blank and the rest were spiked with for PX (1–10 μΜ) and CAF (5–25 μΜ). The samples were treated as previously described, which involved the separation of the analytes from all the proteins and biomolecules present in the saliva samples, and the final solutions were analysed with the optimised DPV method. The first scan was only used each time as the following ones resulted in considerably lower intensities, presumably due to partial blocking of the working surface with co-extracted biocomponents from saliva. Between measurements, the working electrode was repolished to mirror finish. The calibration was repeated three times at three different days and the average charge (*Q*) values were plotted against the final concentrations. This calibration curve was used for quantification of the PX and CAF in human saliva and CYP1A2 phenotyping. The same samples were also measured by UHPLC-UV. 

### 2.6. Clinical Study

This study was approved by Queen Mary Research Ethics committee (Reference QMERC2019/39) and, upon authorisation, a clinical trial with five volunteers was conducted at the Department of Chemical and Pharmaceutical Sciences at the University of Trieste (Italy). Consent forms were signed by all participants and the guidelines for conducting experiments using human biofluids were respected. The application of the proposed method was demonstrated by the analysis of saliva samples from five healthy volunteers (two females and three males), aged between 21 and 29, that did not take regular medication. The females declared that they were not pregnant or did not take oral contraceptives. The volunteers abstained from methylxanthine-containing beverages and food products (coffee, tea, chocolate, cola, etc.) for 24 h prior the experiment to avoid any specific interference. On the day of the experiment, a single 200 mg CAF dose was orally administrated to the subjects. Saliva samples (10 mL) were collected predose, and 1, 3 and 6 h postdose. The experiments were carried out within the same working day of the saliva collection. 

### 2.7. Data Analysis and Statistical Evaluation

The calibration curves (slope and intercept) were obtained and statistically analysed at a 95% confidence interval using OriginPro 8.0 (OriginLab, Northampton, MA, USA) software. Limit of detection (LOD) and quantification (LOQ) were calculated as three and ten times the standard deviation of the intercept divided by the slope of the corresponding curve, respectively. All voltammetric measurements were performed in triplicate (*n* = 3), except of the intraday repeatability study (*n* = 6), which was expressed in terms of relative standard deviation (RSD). The paired t-test was applied to evaluate the significance of the statistical difference for the results obtained by DPV and UHPLC-UV.

## 3. Results

### 3.1. Influence of Supporting Electrolyte on Voltammetric Behavior of PX and CAF

**Figure 2:**
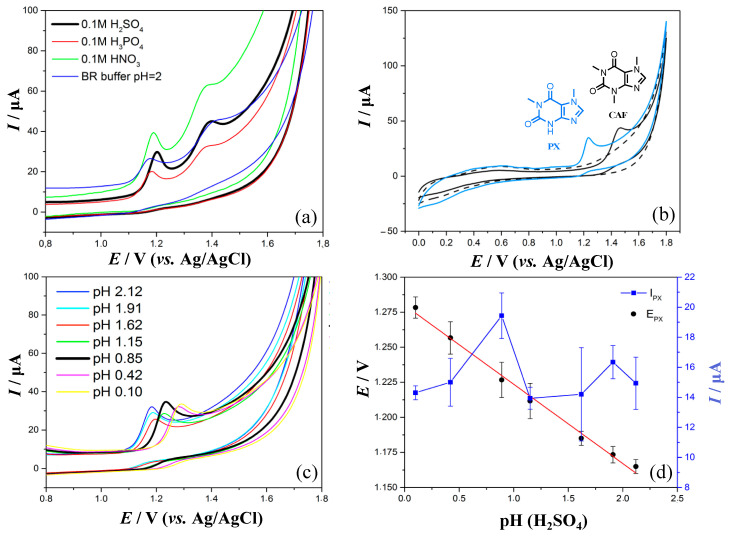
(**a**) Cyclic voltammograms of an equimolar mixture (200 μΜ) of paraxanthine (PX) and caffeine (CAF) in the optimal concentration for the various supporting electrolytes on the glassy carbon (GC) electrode with a scan rate of 100 mV s^−1^; (**b**) cyclic voltammograms of blank 0.1 M H_2_SO_4_ (black dashed line), 500 μM CAF (solid blue line) and 500 μM PX (solid blue line) in 0.1 M H_2_SO_4_ on the GC electrode with a scan rate of 100 mV s^−1^. The chemical structures of the paraxanthine (blue) and caffeine (black) are also given in the figure; (**c**) cyclic voltammograms of 500 μM PX at various pH values of H_2_SO_4_ on the GC electrode with a scan rate of 100 mV s^−1^. The lower lines are the reverse scan of the corresponding upper lines of the same color; (**d**) the effect of pH on the peak potential (*E*) and peak current (*I*) of PX.

The electrochemical response of an equimolar mixture of PX and CAF (0.2 mM) was investigated by carrying out CV experiments (100 mV s^−1^) using an anodically pretreated GC electrode, evaluating H_2_SO_4_, HNO_3_ and H_3_PO_4_ (0.01 to 0.8 M concentration range), as well as Britton–Robinson (BR) buffers (pH 2–10) as supporting electrolytes (Figure S3). Well-defined signals, with high intensity, together with low background current were observed when using electrolytes at pH < 2, consistent with literature data reported for CAF, TP and TB [61,62]. When using BR buffers at pH > 8, a higher background current was observed, caused by discharge of the electrolyte solution (Figure S3). In Figure 2a, the cyclic voltammograms for a mixture with both PX and CAF are shown, displaying the highest signal-to-noise ratio (S/N) in the various supporting electrolytes. These were obtained using, as electrolytes, H_2_SO_4_, HNO_3_ and H_3_PO_4_ at 0.1 M and BR buffer at pH 2. Among these, 0.1 M H_2_SO_4_ showed the best peak resolution and was selected as the electrolyte of choice for the next set of experiments. The individual cyclic voltammograms of PX and CAF solutions at 0.5 mM in 0.1 M H_2_SO_4_, within the potential window from 0.0 V and +1.8 V at a scan rate of 100 mV s^−1^, are shown in Figure 2b. Both analytes display oxidation peaks at different potentials: +1.24 V for PX and +1.46 V for CAF (vs. Ag/AgCl). In the reverse scan using the GC electrode, no reduction peak was observed, suggesting that the electron transfer during the oxidation of PX and CAF is completely irreversible. Although similar behavior has been reported for CAF [57,63], to the best of our knowledge this is the first report of the electrochemical characterisation of PX.

### 3.2. Oxidation Mechanism of Paraxanthine on the GC Electrode

CV experiments can yield significant kinetic and mechanistic information related to the electron transfer mechanism of an oxidation reaction, by investigating the effect of the pH and the scan rate on the peak current (*I*) and peak potential (*E*). First, the influence of the pH on the peak current and potential of 0.5 mM PX was studied by CV, at a scan rate of 100 mV s^−1^, using H_2_SO_4_ in the pH range 0.10 to 2.12 (Figure 2c).

The peak’s current value was not altered significantly as a function of pH in the range investigated (Figure 2d), when the experimental error is taken into account. For the oxidation reaction of PX on the GC electrode, a shift in peak potential of 155 mV to lower values, as illustrated in Figure 2d, was observed when the pH of the electrolyte was increased from 0.10 to 2.12. This provides evidence of the participation of protons in the reaction. However, no significant displacement of the potential was observed as PX is present in its protonated form in the investigated pH range. The relationship between potential and pH was found to be linear and expressed by the following equation:

*E* (V) = 1.28 − 0.056 × pH (1)

The negative slope of the potential vs. pH data indicates the release of protons during the oxidation reaction of PX and its value (−0.056 V pH^−1^) was recognised to be very close to the theoretical value of 0.059 V pH^−1^ for a Nernstian process, revealing that the oxidation of PX on the GC electrode occurs via the overall release of two protons and two electrons [64].

**Scheme 1:**
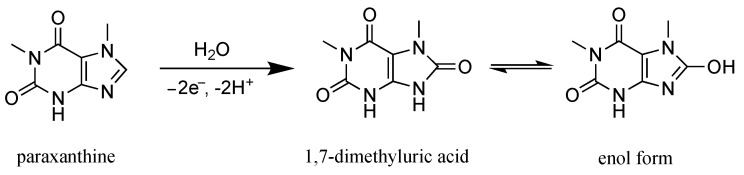
Overall proposed oxidation mechanism of paraxanthine in 0.1 M H_2_SO_4_ on the GC electrode.

Interestingly, the oxidation mechanism of TP, a structural analogue of PX (Figure 1), has also been studied by voltammetry [65,66,67] at various pHs, which can play a critical role in its mechanism [68], and the results were confirmed using other techniques, such as pulse radiolysis [68,69] followed by high resolution mass spectroscopy [68,70,71], UV/Vis spectrophotometry [65,69,72] and computational methods [65,70]. The reported data were used as comparison. The oxidation of TP is a two-electron and two-proton process at pH < 3, with data suggesting the formation of a radical cation as intermediate, resulting in the formation of 1,3-dimethyluric acid. The pK_a_ of H on N3 for PX is 8.5, very similar to the H on N7 of TP (pK_a_ = 8.6) [73,74]. Furthermore, the two molecules also show identical oxidising potential (Figure S13) [62,72]. Given the similarities between PX with TP and the CV data obtained for PX, it can be reasonably concluded that the oxidation of PX in pH < 2 also leads to the formation of 1,7-dimethyluric acid (Scheme 1).

**Figure 3:**
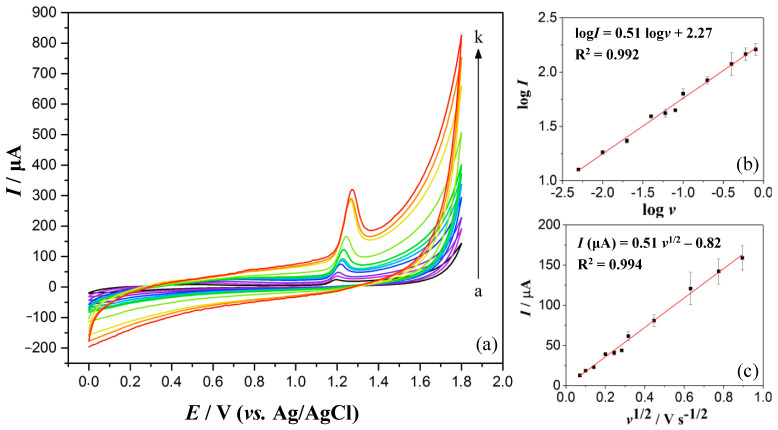
(**a**) Cyclic voltammograms of 400 μM PX at various scan rates (from a to k): 5, 10, 20, 40, 60, 80, 100, 200, 400, 600 and 800 mV s^−1^ in 0.1 M H_2_SO_4_ on the GC electrode; (**b**) log*I* against log*v*; (**c**) *I* against *v*^1/2^.

CV was then used to investigate the effect of scan rate (*v*) on the oxidation of PX (400 μM) in 0.1 M H_2_SO_4_ in the range of 5–800 mV s^−1^ (Figure 3). The oxidising potential shifted to higher values, from 1.195 to 1.270 V (Table S1), with increased scan rate, as expected for an electron transfer, followed by a chemical reaction (EC reaction). A plot of the log*I* vs. log*v* data **(**Figure 3b) showed a good linear fitting with a slope of 0.51, close to the calculated value of 0.50, for a process entirely driven by diffusion [75,76]. This is further confirmed by the linearity of the peak current (*I*) vs. *v*^1/2^ data, with R^2^ = 0.994 (Figure 3c), and the low intercept value (0.82 μA).

In the case of a totally irreversible reaction with a linear dependence of *I* with *v*^1/2^, the electron transfer coefficient (*α*) of the rate determining step for a multi-step electron process can be obtained from the Tafel plot (Figure S4), *E* vs. log*I*, and can be related to the Tafel slope (Equation (S1)) [77]. In the case of PX, the calculated electron transfer coefficient (*α*) was 0.7, which suggests a one-electron process when compared with the theoretical value (0.5 ± 0.2) [78]. 

The number of electrons involved in the rate determining step was confirmed from the plot of *E* vs. ln*v* (Figure S5, Equation (S2)) [77], resulting in *αn* = 0.7 and therefore indicating that *n* = 1. The data confirm that the oxidation of PX is a multi-step electron-transfer process with a rate determining step, defining the electrode’s kinetics, involving a single electron. The next step focused on the quantitative assessment of PX and CAF and DPV was identified as the most sensitive voltammetric technique.

### 3.3. Optimisation of DPV Operating Parameters

The use of DPV for analytical purposes required optimal peak separation between PX and CAF, with the lowest possible background noise, in the range of concentrations found in saliva samples (1–30 μM). Optimisation was carried out using an equimolar mixture of 25 μM PX and CAF in 0.1 M H_2_SO_4_. The pretreatment potential (*E_pret_*) and time (*t_pret_*), scan rate (*v*) and step potential (*E_step_*) were considered essential parameters for evaluation. The effect of electrochemical pretreatment was investigated by application of various anodic (+1 V and +2 V) and cathodic potentials (−2 V and −1 V), and pretreatment times (30, 60 and 120 s) at a fixed step potential of 5 mV and scan rate of 10 mV s^−1^ (Figures S4 and S5). The measurements for the anodically pretreated electrode displayed an increased sensitivity compared to the cathodically pretreated one and the untreated electrode, with +1 V giving the best results in terms of peak separation and height (Figure S6). Changes in the pretreatment time did not improve the signals and it was therefore kept at the lowest value of 30 s (Figure S7). Instead, an increase in the current response was observed with a decrease in the scan rate (Figure S8), and therefore the latter was set at 5 mV s^−1^. Lastly, with a fixed scan rate of 5 mV s^−1^, pretreatment potential +1 V and time of 30 s, the step potential was investigated in the range 1 to 6 mV (Figure S9). Optimal resolution and intensity of the peaks were obtained at a step potential of 5 mV. Thus, the optimised DPV operating parameters were identified as: *E_pret_* of +1 V, *t_pret_* of 30 s, *E_step_* of 5 mV and *v* of 5 mV s^−1^. These experimental conditions were used for the quantification of PX and CAF. 

### 3.4. Quantification of PX and CAF in 0.1 M H_2_SO_4_ by DPV

**Figure 4:**
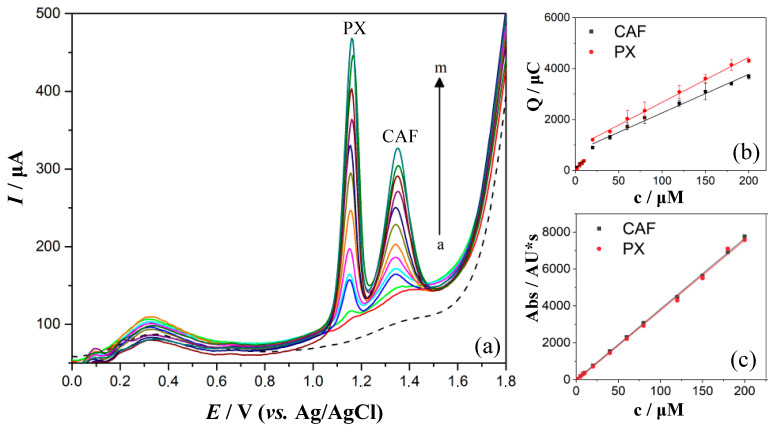
(**a**) DP voltammograms of equimolar mixtures of paraxanthine (PX, *E_PX_* = +1.15 V) and caffeine (CAF, *E_CAF_* = +1.36 V): blank, 1, 2, 5, 8, 20, 40, 60, 80, 120, 150, 180 and 200 μM in 0.1 M H_2_SO_4_ on the GC electrode at a scan rate of 5 mV s^−1^; corresponding calibration curves by (**b**) differential pulse voltammetry (DPV) and (**c**) UHPLC-UV.

All calibration curves were obtained by measuring the charge (peak area, *Q*) as a function of concentration and the data were confirmed by UHPLC-UV. For both analytes, the DPV measurements show a well-defined peak at potential +1.15 V for PX and +1.36 V for CAF in a 0.1 M H_2_SO_4_ solution. The potential difference of 210 mV between the two peaks was sufficiently large to allow simultaneous quantification of the two compounds. The obtained DP voltammograms and the corresponding calibration curves for DPV and UHPLC-UV are shown in Figure 4. 

The peak charges of PX and CAF at +1.16 V and +1.36 V (vs. Ag/AgCl), respectively, increased proportionally with their concentrations up to 200 μM. Higher concentrations were not evaluated, as these would likely result in partial merging of the peaks, considerably limiting the accurate quantification of the analytes. All analytical parameters for their individual and simultaneous quantification by DPV and UHPLC-UV can be found in the supplementary information (Tables S2 and S3). In the case of solutions containing both analytes, linearity of response was observed up to 200 μM using UHPLC-UV. In the case of DPV, two linear segments were observed, the first one up to 10 μM (Figure S10) and the second one between 11 and 200 μM. This previously reported occurrence is likely due to a slight adsorption of the compounds on the surface of the GC electrode, at higher analyte concentrations, and can result in lower sensitivity [79,80,81]. The LOD was found to be 0.59 μM for PX and 0.96 μM for CAF when analysed simultaneously; these values are consistent with literature data for CAF, TP and TB using bare carbon electrodes [61,62,82].

The intraday repeatability of the method gave a relative standard deviation (RSD) of 5.4% and 4.7% for an equimolar mixture of 10 μM PX and CAF, respectively, suggesting good reproducibility of the methodology.

### 3.5. Selectivity Study

Prior to the application of the proposed DPV method for CYP1A2 phenotyping, its selectivity was evaluated. The influence of potential interfering molecules that may be present in saliva was examined with TP and TB, the other two primary metabolites of CAF, which are considered to be the most significant interferents for the target application. The DP voltammograms (Figure S11) showed that TB has identical oxidising potential as CAF, and TP identical to PX, consistent with previous literature data [55,58,61,62].

This could have an impact on the simultaneous quantification of PX and CAF in biological samples. However, literature data report that when CAF is completely metabolised, TB and TP are present in 10.8 and 5.4%, respectively, while PX is present in around 82% [8]. Therefore, their contribution to the peaks of CAF and PX can be considered minimal, within the time frame of 6 h used for this assay, with little effect on the final molar ratios. Other common compounds that can be found in saliva are glucose [47,83], ascorbic acid [84,85] and uric acid [51,85], but they are reported to be oxidised at considerably lower potentials between +0.2 V and +0.6 V and therefore were not considered to impact this study.

### 3.6. Quantification of PX and CAF in Spiked Human Saliva

**Figure 5:**
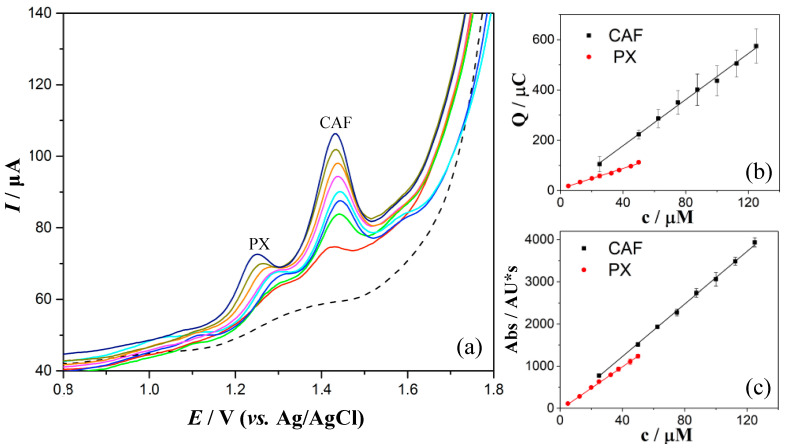
(**a**) DP voltammograms of paraxanthine (PX, *E_PX_* = +1.25 V) and caffeine (CAF, *E_CAF_* = +1.45 V) extracted from spiked saliva on the GC electrode at a scan rate of 5 mV s^−1^; PX and CAF concentrations assuming quantitative extraction: blank saliva (black dashed line), 25 μM and 5 μM (red), 50 μM and 12.5 μM (green), 62.5 μM and 20 μM (blue), 75 μM and 25 μM (light blue), 87.5 μM and 32.5 μM (pink), 100 μM and 37.5 μM (yellow), 112.5 μM and 45 μM (dark yellow), 125 μM and 50 μM (blue); corresponding calibration curves by (**b**) DPV and (**c**) UHPLC-UV.

Literature data report that following consumption of a 100–200 mg CAF dose, the concentrations of PX and CAF found in human saliva 1 h to 6 h postdose are up to 8 μM for PX and 30 μM for CAF [25,34]. This suggested the need for a pre-concentration step of saliva samples that was achieved by a liquid–liquid extraction, providing recovery of around 70–78% for both PX and CAF [33,34,86] and resulting in a five-fold increase in concentration. A new calibration curve in the saliva matrix was obtained with both DPV and UHPLC (Figure 5) using saliva samples from a volunteer and spiking them with PX and CAF solutions. As all the samples are treated to separate the analytes from the saliva biomolecules, the impact of variability of the salivary samples was significantly reduced. Following the established protocol, PX and CAF showed an oxidising potential of +1.25 V and +1.45 V, respectively. A positive shift of around 90 mV of the peak potential for both analytes was observed compared to previous values (Figure 4); the results suggest that the presence of biocomponents co-extracted from saliva impact the oxidation process. All analytical parameters for the simultaneous quantification of PX and CAF in the saliva samples by DPV and UHPLC are presented in Table 1 and Table 2, respectively.

As expected, the LODs by UHPLC were lower than the ones obtained by DPV; however, the simplicity and practicality of DPV mitigates this. The intraday repeatability of the DPV method for binary mixtures of spiked PX (9 μM) and CAF (20 μM) in a saliva sample resulted in RSDs of 7.0% and 4.3%, respectively.

It is of interest to note that experiments using a boron doped diamond (BDD) electrode and screen-printed carbon paste (SP-CP) electrode in both supporting electrolyte solutions or spiked saliva samples showed a significantly lower performance (Figures S12–S15).

### 3.7. Evaluation of Method with Five Volunteers 

**Figure 6:**
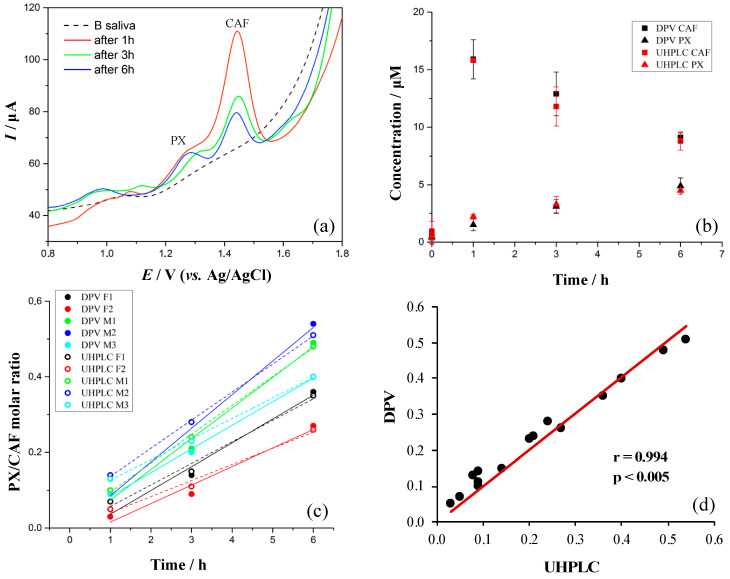
(**a**) DP voltammograms containing paraxanthine (PX, *E_PX_* = +1.24 V) and caffeine (CAF, *E_CA_*_F_ = +1.44 V), isolated from saliva, in 0.1 M H_2_SO_4_ on the GC electrode, with a scan rate of 5 mV s^−1^: predose as blank saliva (B saliva), 1, 3 and 6 h upon administration of a single 200 mg CAF dose from a male volunteer; (**b**) CAF and PX concentration-time profiles by DPV and UHPLC for a male volunteer in three different days upon administration of a single 200 mg CAF dose with SD of the three measurements as error bars; (**c**) PX/CAF molar ratios vs. time (h) by DPV (filled circles and solid lines) and UHPLC-UV (empty circles and dashed lines) for all five subjects (F1 and F2 for females and M1, M2 and M3 for males); (**d**) Correlation plot of PX/CAF molar ratios obtained by DPV vs. UHPLC.

In order to assess the potential of this electrochemical sensing platform, CYP1A2 phenotyping was carried out by monitoring the PX/CAF molar ratio in saliva from five healthy regular coffee drinkers (two females and three males) over time (4 time points; 0–6 h). For the purpose of this study, non-genetic characteristics known to influence the caffeine metabolism, like smoker vs. non-smoker, were not taken into account. A single dose of 200 mg CAF was orally administered to five healthy subjects and saliva samples were collected predose, and 1, 3 and 6 h postdose. The saliva samples were treated as described in the experimental section and analysed with the optimised DPV method and the data were confirmed by UHPLC-UV. Figure 6a shows representative DP voltammograms at the various time intervals from a male volunteer for a single day experiment. 

Typical UHPLC-UV chromatograms obtained for the various time points are shown in Figure S16 (ESI). The concentration of PX and CAF in each sample was calculated using the matrix-matched calibration curve. The concentration profiles (Figure 6a and Figure S17) for PX and CAF and their corresponding molar ratios were obtained for three experiments in three different days per subject, employing the proposed DPV method and UHPLC-UV (Table S4). The concentration profiles with time for a male volunteer are shown in Figure 6b. For all tested subjects, a linear decrease in CAF and increase in PX concentration were observed over time, consistent with the first-order pharmacokinetics of CAF in humans [8,87]. Furthermore, for all tested subjects, a linear increase in the PX/CAF molar ratios over time was observed with both methods, as illustrated in Figure 6c, indicating a proportional increase in the concentration of PX with the decrease in CAF. The molar ratios obtained 1 h postdose by DPV showed the lowest correlation with UHPLC-UV (Table S4), as a result of difficulties in resolving the baseline. However, for CYP1A2 phenotyping, the PX/CAF molar ratio at a single time-point is required. Previous studies in the literature have recommended the use of PX/CAF molar ratios in saliva 4 h and 6 h postdose as a convenient sampling time-point for implementation in a clinical setting [28,34]. In this work, the strongest correlation between the two methods, accurately reflecting the CYP1A2 activity, was found to be 6 h postdose, where the PX/CAF molar ratios ranged from 0.34 to 0.54, consistent with the values reported using HPLC-UV by Jordan et al. and Perera et al. [33,34].

The data demonstrate a high correlation (Pearson correlation coefficient r = 0.994) between the PX/CAF molar ratios values obtained with DPV and UHPLC (Figure 6d). The paired t-test was applied, and the calculated t value (1.74) was lower than the critical value (2.20) at a 95% confidence level, rejecting the null hypothesis. This indicates that the estimated molar ratios by DPV are not statistically significantly different to those measured by UHPLC-UV. This demonstrates that the proposed protocol effectively mitigates the interfering matrix effects of human saliva for the selective determination of the analytes by DPV. 

The proposed sensing system based on DPV is fast, requiring only 6.5 min for the evaluation of each sample. Moreover, the proposed electrochemical system is a portable sensor that is considerably cheaper and easier to operate compared with UHPLC. Although the LOD for the UHPLC-UV is lower compared to DPV, the concentrations of PX detected in human saliva are sufficiently high for estimating the CYP1A2 activity. 

## 4. Conclusions

In this work, we developed a simple and fast analytical tool for the simultaneous quantification of antioxidants CAF and PX in human saliva by DPV on an anodically pretreated GC electrode, for applications in CYP1A2 phenotyping. Voltammetry allowed us to study, for the first time, the oxidation behaviour of PX in aqueous media, revealing that its oxidation occurs via the transfer of two electrons and two protons, similarly to the mechanism reported for TP but very different from CAF. The DPV method was validated via UHPLC-UV, and matrix-matched calibration curves were used for the quantification of PX and CAF in saliva. A limit of detection of 2.89 μM for PX and 5.80 μM for CAF was obtained. Potential applicability of the sensing platform was demonstrated by running a small scale trial on five healthy volunteers, with simultaneous quantification by DPV of PX and CAF in saliva samples collected at 1, 3 and 6 h postdose administration. The results were validated by UHPLC-UV, and were shown to have a high correlation factor (r = 0.994).

The new electrochemical sensor allows the selective and simultaneous quantification of PX and CAF in human saliva with an estimated 20-min total analysis time (from sample collection to voltammogram integration), providing a more accessible tool compared to the standard chromatographic techniques. The precision and accuracy demonstrated makes this approach an attractive alternative and therapeutically useful for monitoring probe drugs and their metabolites in saliva and potentially other biofluids (e.g., sweat and plasma). A higher sensitivity could be achieved by modifying the working electrode, but this would require time-consuming steps, additional costs and potentially lead to lower reproducibility. As a next step, further studies using larger numbers of volunteers will be required to evaluate how the electrochemical sensing platform handles interpatient variations. Our work highlights a range of opportunities for the development of point-of-care devices based on electrochemical sensing for phenotyping drug-metabolising enzymes with clinical significance in the area of personalised medicine. 

## Figures and Tables

**Table 1:** Analytical parameters for the simultaneous quantification of paraxanthine (PX) and caffeine (CAF) in spiked saliva by DPV.

Analytical Parameter	DPV	
	PX	CAF
Sensitivity (μC/μM)	2.02 ± 0.06	4.55 ± 0.10
Intercept (μC)	7.37 ± 1.95	−3.67 ± 8.80
R^2^	0.994	0.996
LOD (μM)	2.89	5.80
Linear range (μM)	10–60	19–125

**Table 2:** Analytical parameters for the simultaneous quantification of paraxanthine (PX) and caffeine (CAF) in spiked saliva by UHPLC-UV.

Analytical Parameter	UHPLC-UV	
	PX	CAF
Sensitivity (AU/μM)	25.01 ± 0.28	31.41 ± 0.41
Intercept (AU)	−14.01 ± 9.01	−39.01 ± 35.01
R^2^	0.999	0.999
LOD (μM)	1.07	3.34
Linear range (μM)	4–60	11–125

## Data Availability

The data presented in this study are available in www.mdpi.com/xxx/s1.

## Supplementary Materials

The following are available online at https://www.mdpi.com/2076-3921/10/1/10/s1. Figure S1: (a) DP voltammograms for increasing concentrations of caffeine keeping paraxanthine constant at 10 μM. Caffeine concentrations: (a) 0, (b) 1, (c) 4, (d) 8, (e) 20, (f) 40, (g) 80, (h) 100, (i) 130, (j) 160, (k) 200, (l) 250 and (m) 300 μM in 0.1 M H_2_SO_4_ on the GC electrode at a scan rate of 5 mV s^−1^; corresponding calibration curves by (b) DPV and (c) UHPLC-UV; Figure S2: (a) Differential pulse voltammograms for increasing concentrations of paraxanthine keeping caffeine constant at 10 μM. Paraxanthine concentrations: (a) 0, (b) 1, (c) 2, (d) 5, (e) 8, (f) 20, (g) 40, (h) 60, (i) 80, (j) 120, (k) 150, (l) 180 and (m) 200 μM in 0.1 M H_2_SO_4_ on the glassy carbon electrode at a scan rate of 5 mV s^−1^; corresponding calibration curves by (b) DPV and (c) UHPLC-UV; Figure S3: Cyclic voltammograms of an equimolar mixture (0.2 mM) of paraxanthine and caffeine from 0.8 to 1.8 V in various concentrations of H_2_SO_4_, HNO_3_, H_3_PO_4_ and Britton–Robinson (BR) buffers on the glassy carbon electrode at a fixed scan rate of 100 mV s^−1^; Figure S4: Tafel plot for paraxanthine: oxidising potential (*E*) against the logarithm of the current (log*I*); Figure S5: Oxidising potential (*E*) against the natural logarithm of the scan rate (ln*v*) for paraxanthine on the glassy carbon electrode; Figure S6: Differential pulse voltammograms of an equimolar mixture (25 μM) of paraxanthine and caffeine in 0.1 M H_2_SO_4_ on the glassy carbon electrode without pretreatment and different pretreatment potentials (*E_PRET_*) with a fixed step potential of 5 mV and scan rate of 10 mV s^−1^; Figure S7: Differential pulse voltammograms of an equimolar (25 μM) mixture of paraxanthine and caffeine in 0.1 M H_2_SO_4_ on a glassy carbon electrode at various pretreatment times: 30, 60 and 120 s with fixed pretreatment potential of +1 V and step potential of 0.005 V at a scan rate of 10 mV s^−1^; Figure S8: Differential pulse voltammograms of an equimolar (25 μM) mixture of paraxanthine and caffeine in 0.1 M H_2_SO_4_ on the glassy carbon electrode at various scan rates: 5, 10, 20, 30, 40, 60 and 100 mV s^−1^; Figure S9: Differential pulse voltammograms of an equimolar (25 μM) mixture of paraxanthine and caffeine in 0.1 M H_2_SO_4_ on the glassy carbon electrode with a pretreatment potential of +1 V and pretreatment time of 30 s for various step potentials (1, 2, 5 and 6 mV) at a scan rate of 5 mV s^−1^; Figure S10: Calibration curve for the simultaneous quantification of equimolar mixtures of paraxanthine (PX) and caffeine (CAF) by differential pulse voltammetry in 0.1 M H_2_SO_4_ on the GC electrode: first linear segment (1–10 μM); Figure S11: Differential pulse voltammograms of an equimolar mixture (50 μM) of caffeine (CAF), paraxanthine (PX), theophylline (TP) and theobromine (TB) in 0.1 M H_2_SO_4_ on the GC electrode, at a scan rate (*v*) of 5 mV s^−1^; Figure S12: Differential pulse voltammograms of 0.1 M H_2_SO_4_ (black dashed line) and an equimolar (50 μM) mixture of paraxanthine (E_PX_ = +1.23 V) and caffeine (E_CAF_ = +1.40 V, red line) on the boron-doped diamond electrode with differential pulse voltammetry parameters: pretreatment potential (*E_PRET_*) of +2 V, pretreatment time (*t_PRET_*) of 30 s, step potential (*E_STEP_*) of 0.005 V, pulse potential (*E_PULS_*) of 0.1 V, pulse time (*t_PULS_*) of 10 ms and scan rate (*v*) of 100 mV s^−1^; Figure S13: Differential pulse voltammograms for 0.01 M H_2_SO_4_ (black dashed line), treated blank saliva (red line), treated spiked water (green line) and treated spiked saliva (blue line) with equimolar concentrations (100 μM) caffeine and paraxanthine on the boron doped diamond electrode; Figure S14: Differential pulse voltammograms of 0.1 M H_3_PO_4_ (black dashed line) and an equimolar mixture (100 μM) of paraxanthine (*E_PX_* = +0.82 V) and caffeine (*E_CAF_* = +1.01 V, red line) on a screen-printed carbon paste electrode with differential pulse voltammetry parameters: step potential (*E_STEP_*) of 0.004 V, pulse potential (*E_PULS_*) of 0.15 V, pulse time (*t_PULS_*) of 10 ms and scan rate (*v*) of 10 mV s^−1^; Figure S15: Differential pulse voltammograms (DPV) for 0.01 M H_2_SO_4_ (black dashed line), extracted caffeine and paraxanthine from saliva 2 h postdose (red line) and 4h postdose (green line); on the screen-printed carbon paste electrode with DPV parameters: step potential of 0.004 V, pulse potential of 0.15 V, pulse time of 10 ms and scan rate of 10 mV s^−1^; Figure S16: UHPLC-UV chromatograms from a volunteer (abstinence from caffeine for 24 h) for a one day experiment obtained upon administration of 200 mg caffeine oral dose: predose (blue), 1 h (red), 3 h (green) and 6 h (pink) postdose; Figure S17: Caffeine (CAF) and paraxanthine (PX) concentration-time profiles by DPV and UHPLC-UV for the rest four tested subjects (F1 and F2 for females and M1 and M2 for males) in three different days upon administration of a single 200 mg CAF dose with standard deviation of the three measurements as error bars. Table S1: Averaged values of peak potential (*E*) and peak current (*I*) for the various scan rates for 400 μM paraxanthine (PX) in 0.1 M H_2_SO_4_ by cyclic voltammetry on the glassy carbon electrode; Table S2: Analytical parameters for the individual and simultaneous determination of paraxanthine and caffeine by differential pulse voltammetry in 0.1 M H_2_SO_4_ on the glassy carbon electrode; Table S3: Analytical parameters for individual and simultaneous determination of paraxanthine and caffeine by UHPLC-UV; Table S4: Human saliva sample analysis for five volunteers at three different days using the proposed DPV method and UHPLC-UV as reference method. Equations (S1) and (S2) were used to calculate the electron transfer coefficient (*α*) and the number of electrons involved in the rate determining step of the oxidation of paraxanthine.

## Abbreviations

IUPAC: International Union of Pure and Applied Chemistry; LOD, limit of detection; LOQ, limit of quantification; RSD, relative standard deviation; GC, glassy carbon; BDD, boron-doped diamond; SP-CP, screen-printed carbon paste; CV, cyclic voltammetry; DPV, differential pulse voltammetry; CYP, P450 cytochromes; PX, paraxanthine; CAF, caffeine; TP, theophylline; TB, theobromine; BR, Britton–Robinson; UHPLC, ultra-high performance liquid chromatography; UV, ultraviolet; MS, mass spectrometry.
