# Supplementary material for: Simultaneous Quantification of Antioxidants Paraxanthine and Caffeine in Human Saliva by Electrochemical Sensing for CYP1A2 Phenotyping
Source: Antioxidants (Basel). 2020 Dec 24;10(1):10. doi: 10.3390/antiox10010010 (PMC7823619; doi:10.3390/antiox10010010)
Supplement: Supplementary file 1 [file antioxidants-10-00010-s001.pdf]

SUPPLEMENTARY INFORMATION

Simultaneous Quantification of Antioxidants Paraxanthine and Caffeine in Human Saliva by Electrochemical Sensing for CYP1A2 Phenotyping

Rozalia-Maria Anastasiadi [1,*], Federico Berti [2], Silvia Colomban [3], Claudio Tavagnacco [2], Luciano Navarini [3] and Marina Resmini [1,*]

[1] Department of Chemistry, Queen Mary University of London, Mile End Road, London E1 4NS, UK
[2] Department of Chemical and Pharmaceutical Sciences, University of Trieste, via L. Giorgieri 1, 34127 Trieste, Italy; fberti@units.it (F.B.); c.tavagnacco@units.it (C.T.)
[3] Aromalab, illycaffè S.p.A., Area Science Park, Localita' Padriciano 99, 34149 Trieste, Italy; silvia.colomban@illy.com (S.C.); luciano.navarini@illy.com (L.N.)
* Correspondence: rozalia-maria.anastasiadi@utc.fr (R.-M.A.); m.resmini@qmul.ac.uk (M.R.)

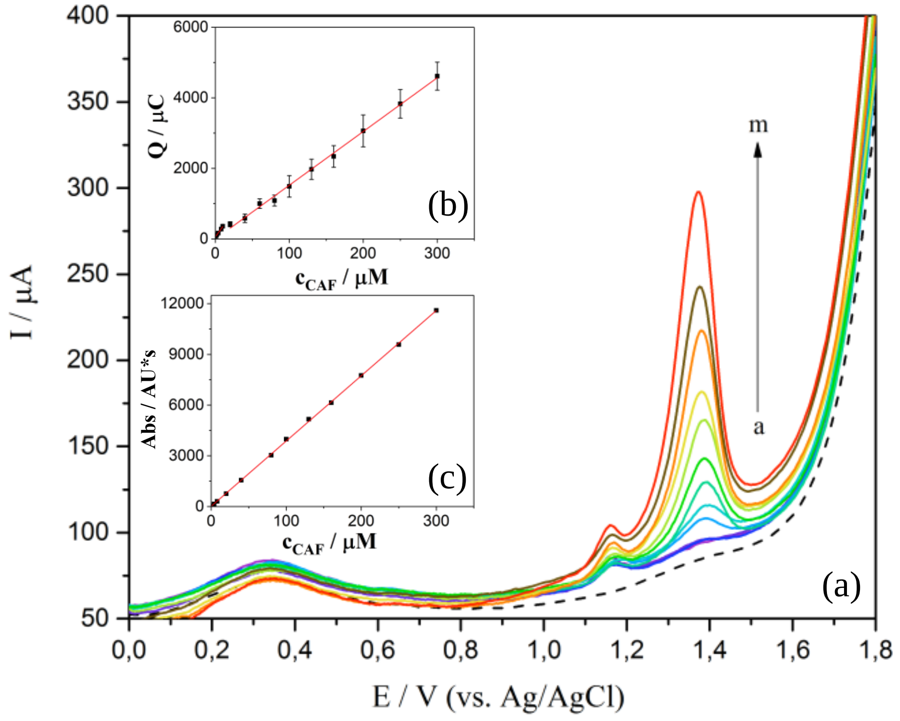

Figure S1. (a) DP voltammograms for increasing concentrations of caffeine keeping paraxanthine constant at 10 µM. Caffeine concentrations: (a) 0, (b) 1, (c) 4, (d) 8, (e) 20, (f) 40, (g) 80, (h) 100, (i) 130, (j) 160, (k) 200, (l) 250 and (m) 300 µM in 0.1 M H_2SO_4 on the GC electrode at a scan rate of 5 mV s^{-1}; corresponding calibration curves by (b) DPV and (c) UHPLC-UV.

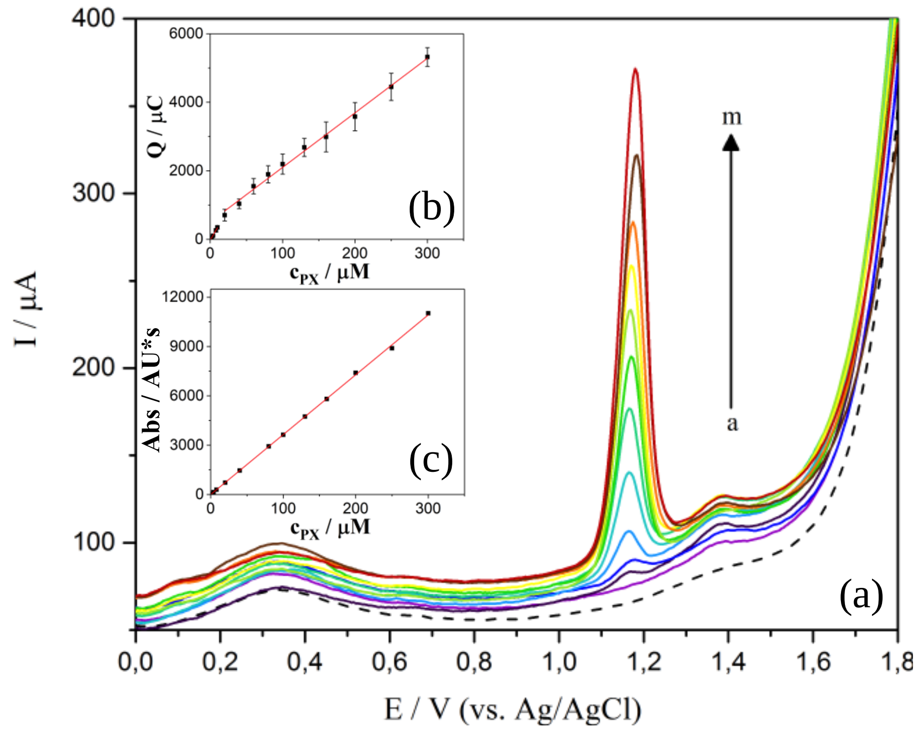

Figure S2. (a) Differential pulse voltammograms for increasing concentrations of paraxanthine keeping caffeine constant at 10 µM. Paraxanthine concentrations: (a) 0, (b) 1, (c) 2, (d) 5, (e) 8, (f) 20, (g), 40, (h) 60, (i) 80, (j) 120, (k) 150, (l) 180 and (m) 200 µM in 0.1 M H_2SO_4 on the glassy carbon electrode at a scan rate of 5 mV s^{-1}; corresponding calibration curves by (b) DPV and (c) UHPLC-UV.

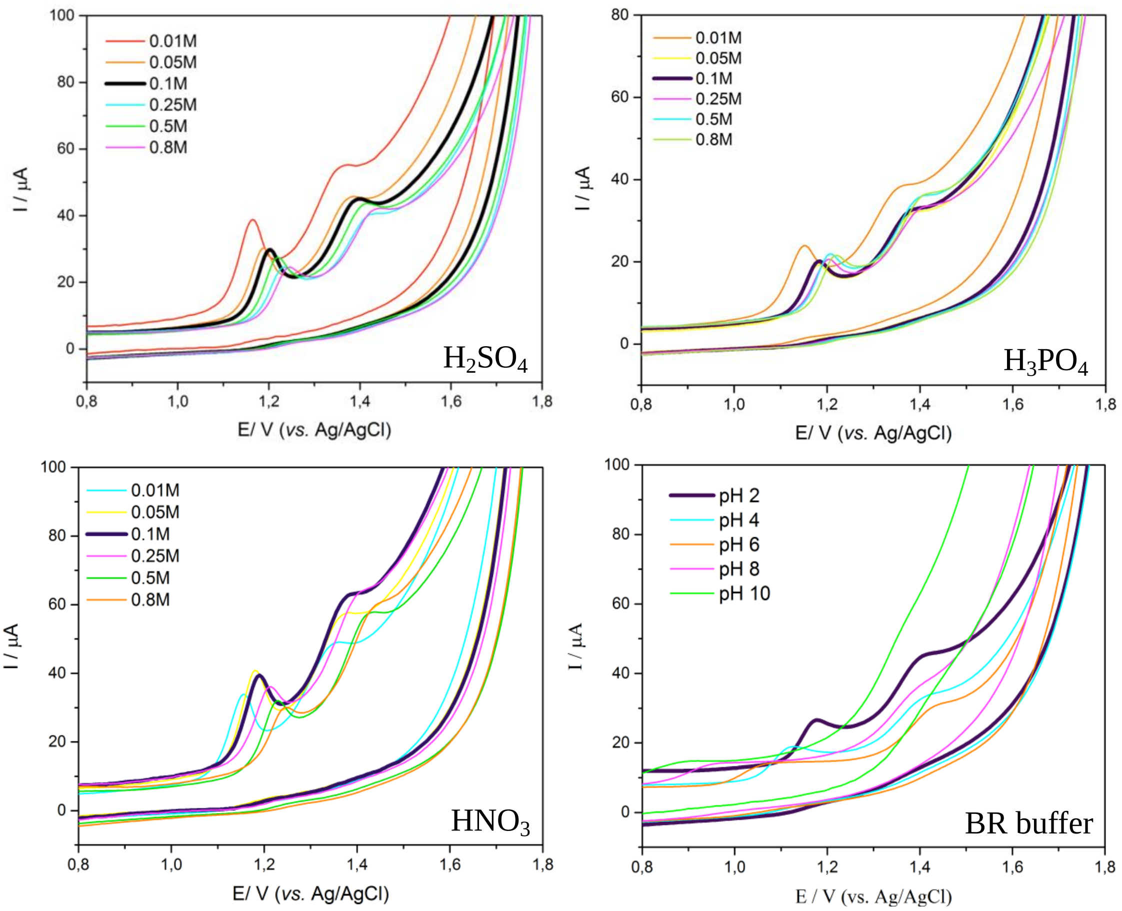

Figure S3. Cyclic voltammograms of an equimolar mixture (0.2 mM) of paraxanthine and caffeine from 0.8 to 1.8 V in various concentrations of H_2SO_4, HNO_3, H_3PO_4 and Britton-Robinson (BR) buffers on the glassy carbon electrode at a fixed scan rate of 100 mV s^{-1}.

Table S1. Averaged values of peak potential (*E*) and peak current (*I*) for the various scan rates for 400 μM paraxanthine (PX) in 0.1 M H_2SO_4 by cyclic voltammetry on the glassy carbon electrode.

Scan rate (mV s^{-1})	E_{PX} (V)	I_{PX} (μA)
**5**	1.18	12.7
**10**	1.19	18.6
**20**	1.20	23.0
**40**	1.21	39.2
**60**	1.22	40.9
**80**	1.22	43.8
**100**	1.23	61.7
**200**	1.24	80.9
**400**	1.26	120.8
**600**	1.27	142.2
**800**	1.27	158.9

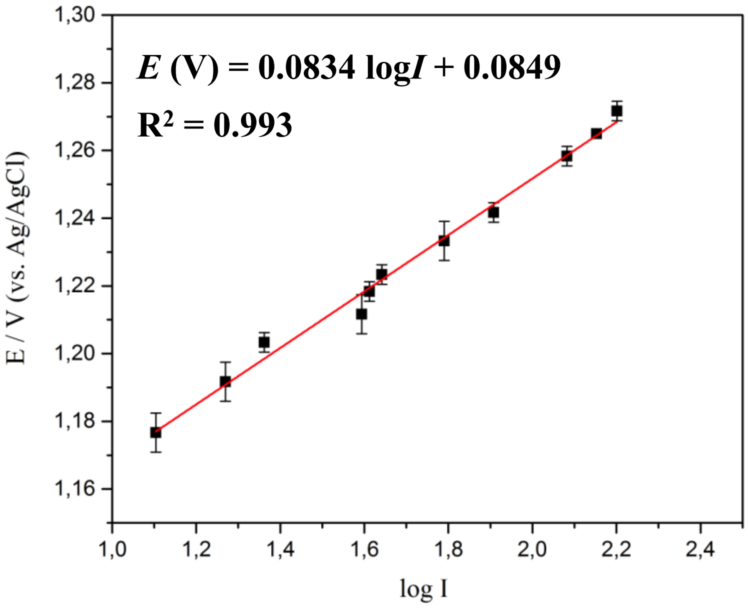

Figure S4. Tafel plot for paraxanthine: oxidising potential (*E*) against the logarithm of the current (log*I*).

The following equations were used to calculate the electron transfer coefficient (α) and the number of electrons involved in the rate determining step of the oxidation of paraxanthine

$$a = {}^{2.3\,RT}/_{F} \times Tafel\ slope \qquad \text{(Eq. S1)}$$

$$slope = {}^{RT}/_{2anF} \qquad \text{(Eq. S2)}$$

where R denotes the gas constant (8.314 J $mol^{-1}K^{-1}$), F the Faraday constant (96485 C mol^{-1}) and T the absolute temperature (298.15 K). Here, the Tafel plot for PX is given by the equation

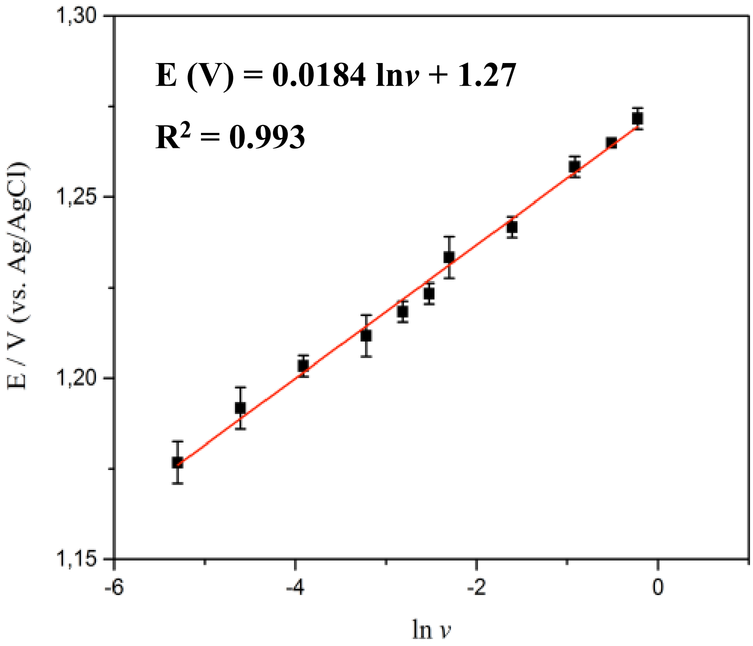

Figure S5. Oxidising potential (E) against the natural logarithm of the scan rate (ln$v$) for paraxanthine on the glassy carbon electrode.

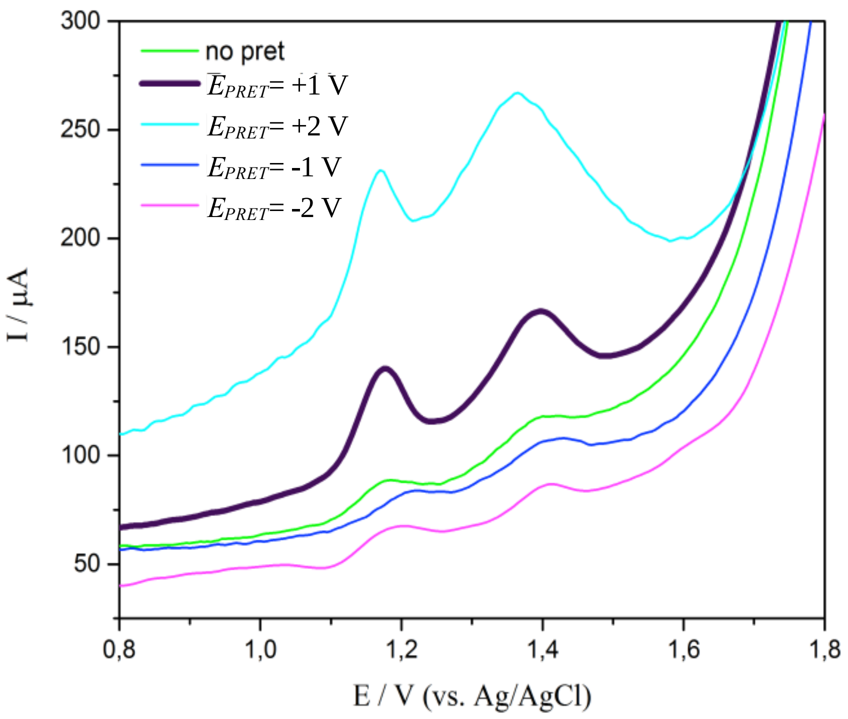

Figure S6. Differential pulse voltammograms of an equimolar mixture (25 µM) of paraxanthine and caffeine in 0.1 M H_2SO_4 on the glassy carbon electrode without pretreatment and different pretreatment potentials (E_{PRET}) with a fixed step potential of 5 mV and scan rate of 10 mV s^{-1}.

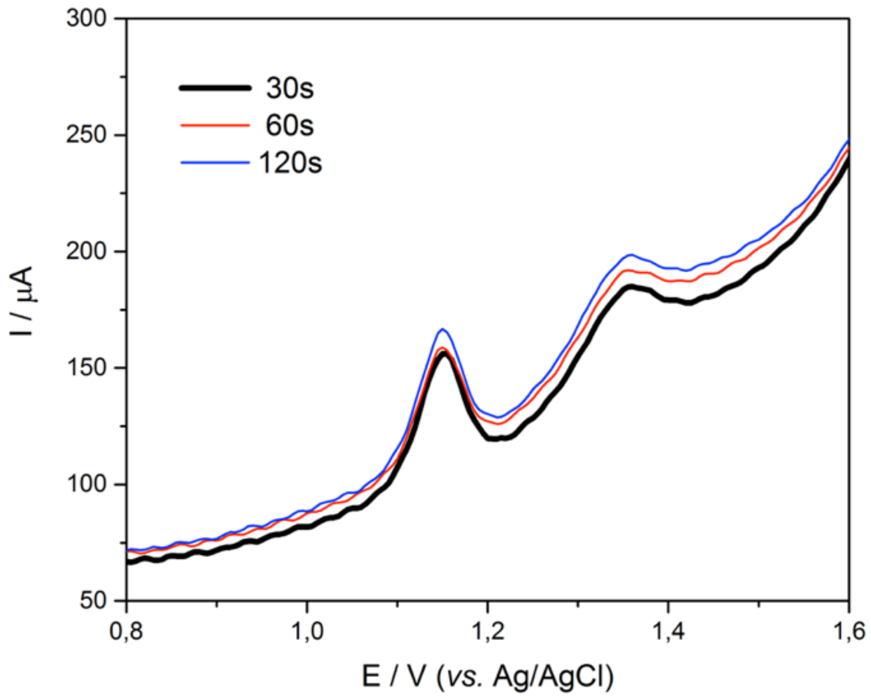

Figure S7. Differential pulse voltammograms of an equimolar (25 µM) mixture of paraxanthine and caffeine in 0.1 M H_2SO_4 on a glassy carbon electrode at various pretreatment times: 30, 60 and 120 s with fixed pretreatment potential of +1 V and step potential of 0.005 V at a scan rate of 10 mV s^{-1}.

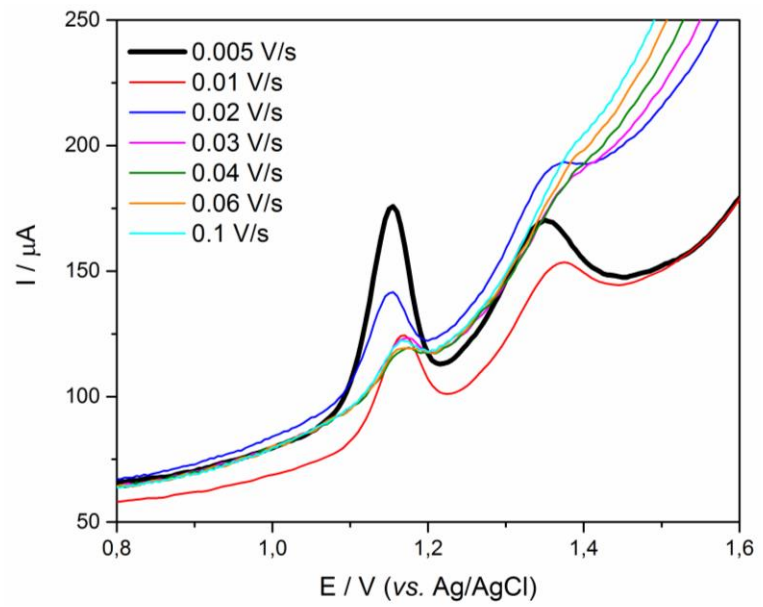

Figure S8. Differential pulse voltammograms of an equimolar (25 µM) mixture of paraxanthine and caffeine in 0.1 M H_2SO_4 on the glassy carbon electrode at various scan rates: 5, 10, 20, 30, 40, 60 and 100 mV s^{-1}.

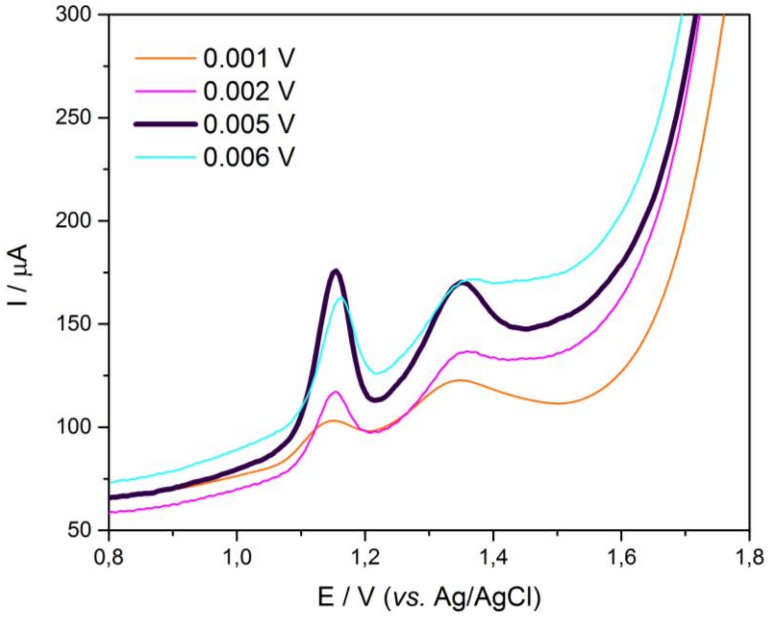

Figure S9. Differential pulse voltammograms of an equimolar (25 µM) mixture of paraxanthine and caffeine in 0.1 M H_2SO_4 on the glassy carbon electrode with a pretreatment potential of +1 V and pretreatment time of 30 s for various step potentials (1, 2, 5 and 6 mV) at a scan rate of 5 mV s^{-1}.

Table S2. Analytical parameters for the individual and simultaneous determination of paraxanthine and caffeine by differential pulse voltammetry in 0.1 M H_2SO_4 on the glassy carbon electrode.

Analytical parameter	Individual determination		Simultaneous determination	
	Paraxanthine	Caffeine	Paraxanthine	Caffeine
Sensitivity (µC/µM)	36.23 ± 1.76 [a]<br>15.93 ± 0.38 [b]	29.55 ± 1.48 [a]<br>15. 23 ± 0.29 [b]	39.09 ± 1.23 [a]<br>17.72 ± 0.47 [b]	30.72 ± 1.59 [a]<br>15.15 ± 0.60 [b]
Intercept (µC)	-24.94 ± 10.73 [a]<br>505.93 ± 60.23 [b]	53.76 ± 9.0 [a]<br>-1.35 ± 46.27 [b]	-9.40 ± 7.66 [a]<br>898.73 ± 58.02 [b]	59.92 ± 9.87 [a]<br>742.95 ± 74.09 [b]
R^2	0.991<br>0.995	0.990<br>0.997	0.996<br>0.995	0.990<br>0.990
LOD[c] (µM)	0.89	0.91	0.59	0.96
Linear range (µM)	3-300<br>(3-10,<br>11-300)	3-300<br>(3-10<br>11-300)	2-200<br>(2-10<br>11-200)	3-200<br>(3-10<br>11-200)
Intra-day repeatability (RSD%)	6.1	4.2	5.4	4.7

[a] 1st segment; [b] 2nd segment; [c] LOD was calculated as 3 x standard deviation/sensitivity (3σ); [d] RSD was calculated for 6 replicates at 10 µM; LOQ was calculated as 10 x standard deviation/sensitivity (3σ); Data presented as mean ± SD.

Table S3. Analytical parameters for individual and simultaneous determination of paraxanthine and caffeine by UHPLC-UV.

Analytical parameter	Individual determination		Simultaneous determination	
	Paraxanthine	Caffeine	Paraxanthine	Caffeine
Sensitivity (mAU/µM)	36.41 ± 0.24	38.61 ± 0.20	35.98 ± 0.17	38.30 ± 0.25
Intercept (mAU)	1.38 ± 35.15	14.24 ± 29.17	7.25 ± 62.38	-2.83 ± 24.73
R^2	0.999	0.999	0.999	0.999
LOD[c] (µM)	0.11	0.10	0.11	0.10
Linear range (µM)	0.3-300	0.3-300	0.3-200	0.3-200

[c] LOD was calculated as S/N ratio; LOQ was calculated as 10 x standard deviation/sensitivity (3σ); Data presented as mean ± SD.

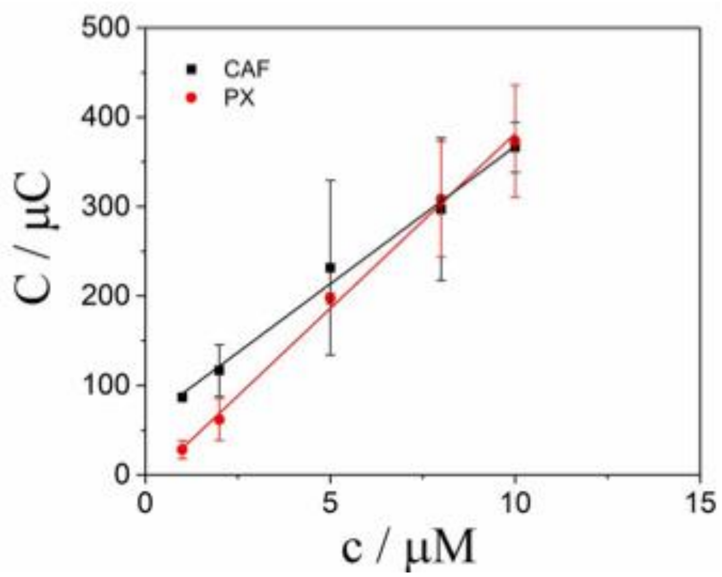

Figure S10. Calibration curve for the simultaneous quantification of equimolar mixtures of paraxanthine (PX) and caffeine (CAF) by differential pulse voltammetry in 0.1 M H_2SO_4 on the GC electrode: first linear segment (1 – 10 μM).

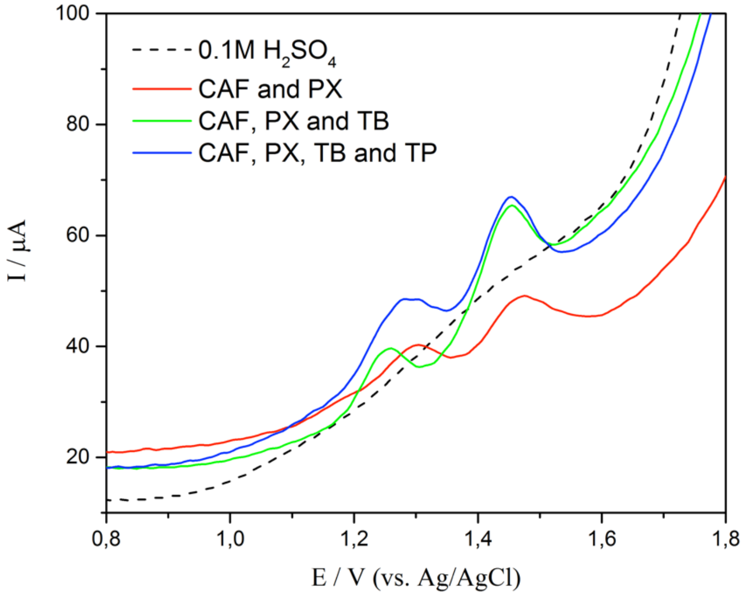

Figure S11. Differential pulse voltammograms of an equimolar mixture (50 μM) of caffeine (CAF), paraxanthine (PX), theophylline (TP) and theobromine (TB) in 0.1 M H_2SO_4 on the GC electrode, at a scan rate (v) of 5 mV s^{-1}.

Comparison with other commercial electrodes. The suitability of a boron doped diamond (BDD) electrode and a carbon paste screen printed (CP-SP) electrode was also evaluated in supporting electrolyte solutions and for the saliva sample analysis. An equimolar mixture (50 µM) of PX and CAF in 0.1 M H_2SO_4 was oxidized at +1.23 and +1.40 V (vs. Ag/AgCl), respectively, using the BDD electrode (Figure S10). However, both compounds were not baseline resolved and that can lead to poor analytical performance. Similarly, upon extraction of the analytes from saliva the resulting peaks were completely merged (Figure S11) not allowing simultaneous determination of the analytes. In the case of the CP-SP, PX and CAF (100 µM) in 0.1 M H_3PO_4 were oxidized at +0.82 V and +1.01 V (Figure S12), respectively, obtaining a good peak separation. However, the current sensitivity was insufficient for concentrations below 30 µM, which is the concentration range required for CYP1A2 phenotyping. Moreover, the detection of the analytes upon saliva pretreatment was not possible given the extremely high background current (Figure S13), presumably due to partial blocking of the electrode's surface by other co-extracted components. Hence, the performance of the GC electrode was proved to be superior for the simultaneous determination of PX and CAF compared with the BDD and CP-SP electrodes.

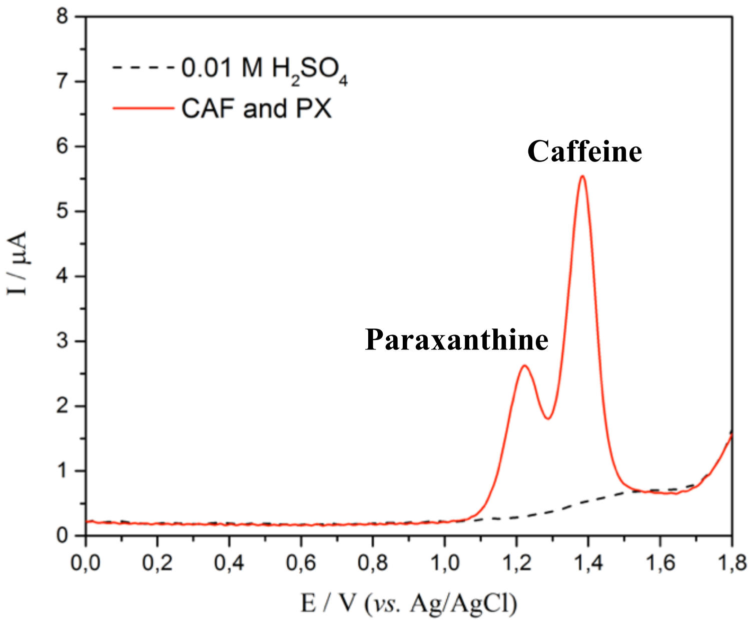

Figure S12. Differential pulse voltammograms of 0.1 M H_2SO_4 (black dashed line) and an equimolar (50 µM) mixture of paraxanthine (E_{PX} = +1.23 V) and caffeine (E_{CAF} = +1.40 V, red line) on the boron-doped diamond electrode with differential pulse voltammetry parameters: pretreatment potential (E_{PRET}) of +2 V, pretreatment time (t_{PRET}) of 30 s, step potential (E_{STEP}) of 0.005 V, pulse potential (E_{PULS}) of 0.1 V, pulse time (t_{PULS}) of 10 ms and scan rate (v) of 100 mV s^{-1}.

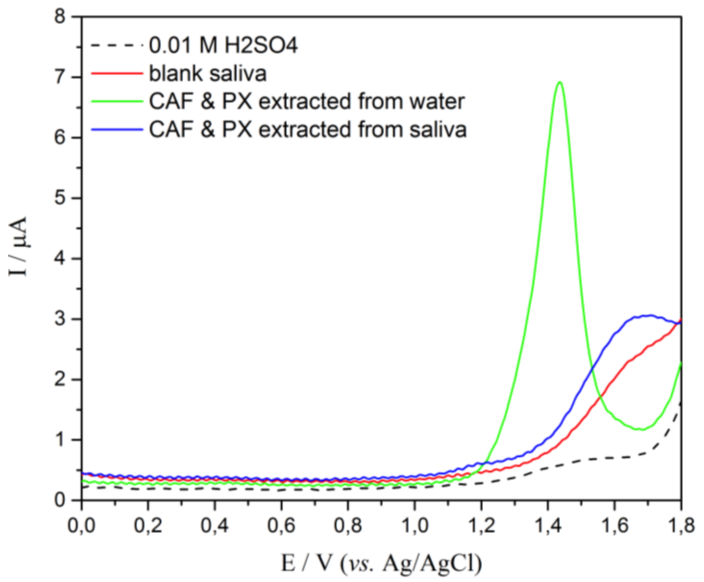

Figure 13. Differential pulse voltammograms for 0.01 M H_2SO_4 (black dashed line), treated blank saliva (red line), treated spiked water (green line) and treated spiked saliva (blue line) with equimolar concentrations (100 µM) caffeine and paraxanthine on the boron doped diamond electrode.

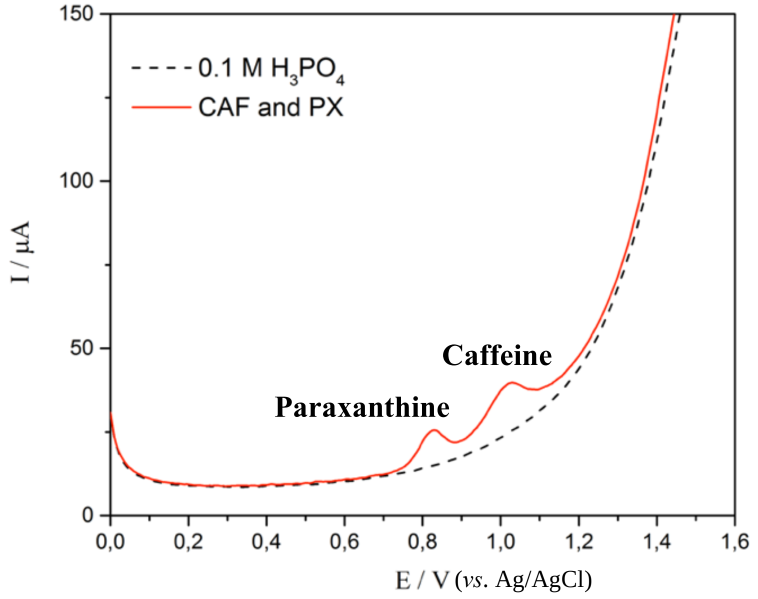

Figure S14. Differential pulse voltammograms of 0.1 M H_3PO_4 (black dashed line) and an equimolar mixture (100 µM) of paraxanthine (E_{PX} = +0.82 V) and caffeine (E_{CAF} = +1.01 V, red line) on a screen-printed carbon paste electrode with differential pulse voltammetry parameters: step potential (E_{STEP}) of 0.004 V, pulse potential (E_{PULS}) of 0.15 V, pulse time (t_{PULS}) of 10 ms and scan rate (v) of 10 mV s^{-1}.

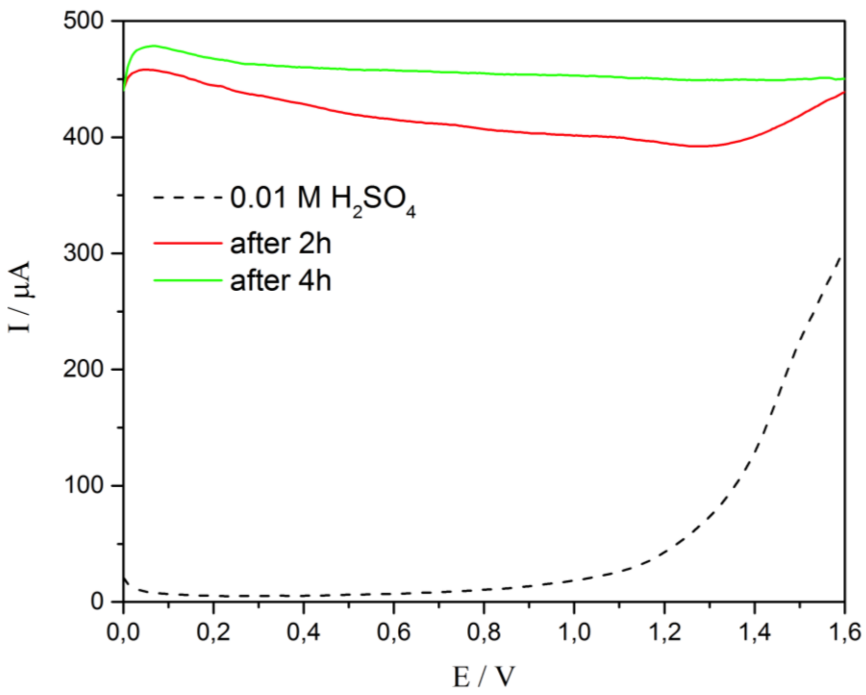

Figure S15. Differential pulse voltammograms (DPV) for 0.01 M H_2SO_4 (black dashed line), extracted caffeine and paraxanthine from saliva 2 h postdose (red line) and 4h postdose (green line); on the screen printed carbon paste electrode with DPV parameters: step potential of 0.004 V, pulse potential of 0.15 V, pulse time of 10 ms and scan rate of 10 mV s^{-1}.

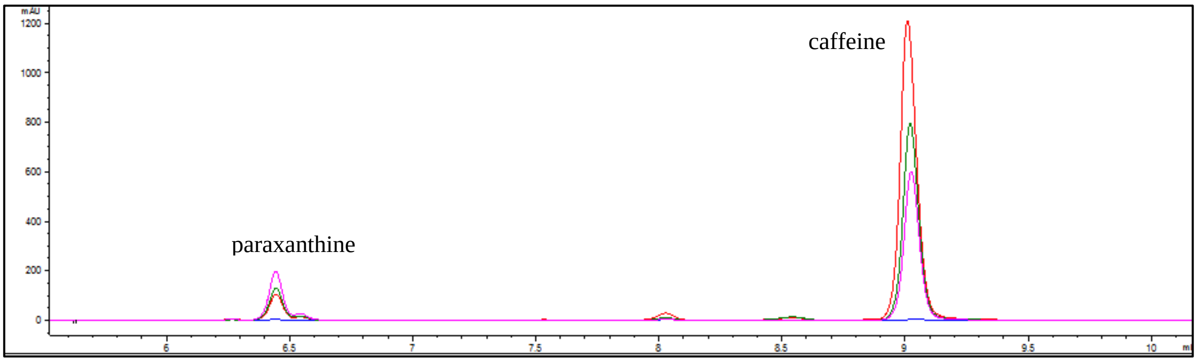

Figure S16. UHPLC-UV chromatograms from a volunteer (abstinence from caffeine for 24 h) for a one day experiment obtained upon administration of 200 mg caffeine oral dose: predose (blue), 1 h (red), 3 h (green) and 6 h (pink) postdose.

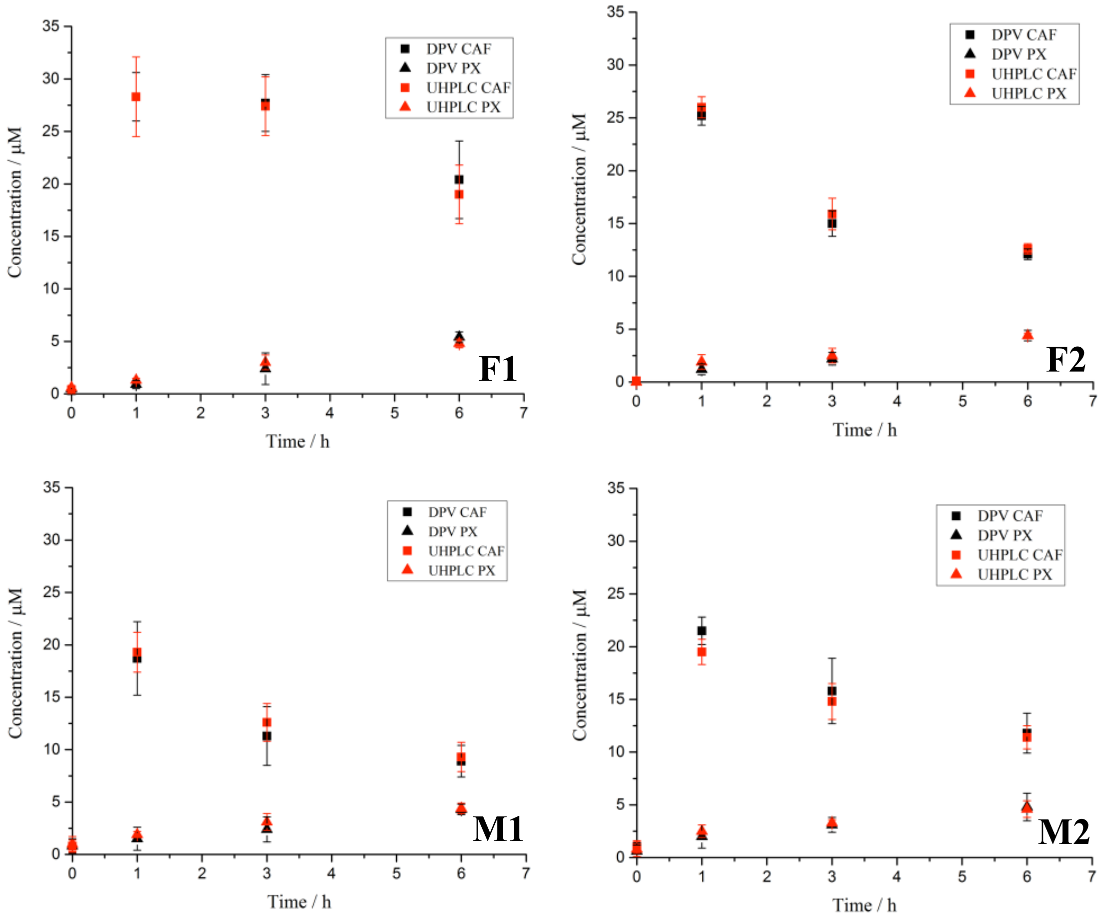

Figure S17. Caffeine (CAF) and paraxanthine (PX) concentration-time profiles by DPV and UHPLC-UV for the rest four tested subjects (F1 and F2 for females and M1 and M2 for males) in three different days upon administration of a single 200 mg CAF dose with standard deviation of the three measurements as error bars.

Table S4. Human saliva sample analysis for five volunteers at three different days using the proposed DPV method and UHPLC-UV as reference method.

		DPV			UHPLC		
		**CAF (µM)**	**PX (µM)**	**[PX]/[CAF]**	**CAF (µM)**	**PX (µM)**	**[PX]/[CAF]**
F1	Pre-dose	ND	ND	NA	0.1 ± 0.1	0.0 ± 0.1	0.0
	1 h	25.2 ± 0.9	1.2 ± 0.5	0.05 ± 0.02	26.0 ± 1.0	1.9 ± 0.7	0.07 ± 0.02
	3 h	15.0 ± 1.2	2.2 ± 0.6	0.14 ± 0.03	15.9 ± 1.5	2.5 ± 0.7	0.15 ± 0.03
	6 h	12.1 ± 0.6	4.4 ± 0.5	0.36 ± 0.03	12.6 ± 0.5	4.4 ± 0.2	0.35 ± 0.01
F2	Pre-dose	0.3 ± 0.1	0.5 ± 0.1	1.42 ± 0.22	0.4 ± 0.2	0.6 ± 0.2	1.56 ± 0.42
	1 h	28.3 ± 2.3	0.8 ± 0.5	0.03 ± 0.01	28.3 ± 3.8	1.3 ± 0.2	0.05 ± 0.01
	3 h	27.7 ± 2.7	2.4 ± 1.5	0.09 ± 0.03	27.4 ± 2.8	3.0 ± 0.7	0.11 ± 0.03
	6 h	20.4 ± 3.7	5.4 ± 0.5	0.27 ± 0.06	19.0 ± 2.8	4.8 ± 0.4	0.26 ± 0.06
M1	Pre-dose	0.8 ± 0.7	0.7 ± 0.7	1.0 ± 0.04	0.8 ± 0.6	1.0 ± 0.7	1.15 ± 0.15
	1 h	18.7 ± 3.5	1.5 ± 1.1	0.09 ± 0.07	19.3 ± 1.9	1.9 ± 0.3	0.10 ± 0.02
	3 h	11.3 ± 2.8	2.4 ± 1.2	0.21 ± 0.06	12.6 ± 1.8	3.1 ± 0.8	0.24 ± 0.03
	6 h	8.9 ± 1.5	4.3 ± 0.5	0.49 ± 0.05	9.3 ± 1.4	4.4 ± 0.5	0.48 ± 0.03
M2	Pre-dose	0.6 ± 0.5	0.4 ± 0.5	0.72 ± 0.40	0.9 ± 0.9	0.5 ± 0.7	0.62 ± 0.42
	1 h	15.9 ± 1.7	1.4 ± 0.6	0.10 ± 0.03	15.8 ± 0.2	2.2 ± 0.2	0.14 ± 0.02
	3 h	12.9 ± 1.9	3.1 ± 0.6	0.24 ± 0.05	11.8 ± 1.7	3.3 ± 0.7	0.28 ± 0.04
	6 h	9.1 ± 0.4	4.9 ± 0.7	0.54 ± 0.06	8.8 ± 0.8	4.5 ± 0.1	0.51 ± 0.05
M3	Pre-dose	0.9 ± 0.5	0.6 ± 0.6	0.58 ± 0.40	1.0 ± 0.6	0.8 ± 0.7	0.68 ± 0.37
	1 h	21.5 ± 1.3	1.6 ± 1.4	0.08 ± 0.07	19.5 ± 1.2	2.5 ± 0.6	0.13 ± 0.04
	3 h	15.8 ± 3.1	3.1 ± 0.7	0.20 ± 0.06	14.8 ± 1.7	3.3 ± 0.3	0.23 ± 0.03
	6 h	11.8 ± 1.9	4.8 ± 1.3	0.40 ± 0.05	11.4 ± 1.1	4.6 ± 0.8	0.51 ± 0.03
